# Venous microtrauma associated with pharmacomechanical catheter-directed thrombolysis in a large animal acute deep venous thrombosis model

**DOI:** 10.3389/fcvm.2025.1567342

**Published:** 2025-06-16

**Authors:** Mohamed S. Zaghloul, Sophia R. Pyeatte, Shahab Hafezi, Usama Ismail, Rodrigo Meade, Batool Arif, Roger Rowe, Luis Sanchez, Mohamed A. Zayed

**Affiliations:** ^1^ Department of Surgery, Section of Vascular Surgery, Washington University School of Medicine, St. Louis, MO, United States; ^2^ Department of Surgery, CardioVascular Research Innovation in Surgery & Engineering Center, Washington University School of Medicine, St. Louis, MO, United States; ^3^ Caeli Vascular, Inc., St. Louis, MO, United States; ^4^ Department of Surgery, Veterans Affairs St. Louis Health Care System, St. Louis, MO, United States; ^5^ Department of Radiology, Washington University School of Medicine, St. Louis, MO, United States; ^6^ Division of Molecular Cell Biology, Washington University School of Medicine, St. Louis, MO, United States; ^7^ Department of Biomedical Engineering, Washington University, McKelvey School of Engineering, St. Louis, MO, United States; ^8^ Department of Surgery, Division of Surgical Sciences and Innovation, Washington University School of Medicine, St. Louis, MO, United States

**Keywords:** deep venous thrombosis, pharmacomechanical catheter-directed thrombolysis, angiojet rheolytic thrombectomy, large animal model, venous wall injury

## Abstract

**Background:**

Pharmacomechanical catheter-directed thrombolysis (PCDT) is commonly used to treat acute deep venous thrombosis (DVT). The AngioJet Rheolytic Thrombectomy (ART) device is a widely used option. However, previous serious adverse events led to an FDA black-box warning. Limited large animal studies have assessed ART's efficacy and safety. We investigated the periprocedural outcomes and venous histomorphic impact of ART in a novel large animal acute DVT model.

**Methods:**

Six adult male Yorkshire pigs (96–113 kilograms) underwent general anesthesia and continuous hemodynamic monitoring. Acute DVT was induced in the infrarenal IVC using occlusive balloons. Three pigs were treated with ART's thrombectomy mode (MT) for 10 min. Another three pigs underwent PCDT with 250 ml saline and 50,000 IU Streptokinase over 10 min. Serial venograms assessed thrombus formation and post-treatment results, and then iliocaval venous segments were resected for histomorphic analysis.

**Results:**

ART significantly reduced thrombus in all pigs without complications (64.7% ± 9.7% vs. 16.1% ± 10.5%; *P* *=* *0.02*). Retroperitoneal staining was observed around treated venous segments in all pigs, with a higher mean staining score in the PCDT group compared to the MT group (2 ± 0 vs. 1.3 ± 0.57; *P* *=* *0.11*)*.* Histopathology revealed more venous wall intimal tears in ART-treated segments compared to untreated segments (18 ± 5.79 vs. 6.3 ± 2.51, *P* *=* *0.01*).

**Conclusions:**

ART effectively removes acute thrombus in a large animal DVT model without periprocedural complications. The observed retroperitoneal staining following PCDT raises some concerns regarding venous wall integrity following ART, for which the long-term consequences are unknown.

## Introduction

Deep Vein Thrombosis (DVT) is a significant public health concern, with an annual incidence of >900,000 in the US and up to 10 million worldwide (1). A burden of 7–12 billion dollars is placed on the American healthcare system each year due to the treatment of DVT and other venous thromboembolism (VTE) events (2). Anticoagulation therapy is highly effective in managing acute DVT, including distal venous structures. However, managing large-volume DVT, particularly in the iliocaval system, presents additional challenges where anticoagulation alone may not be sufficient to restore venous patency or prevent complications such as post-thrombotic syndrome (3). Accordingly, multiple thrombectomy device pivotal studies have demonstrated that operative thrombectomy of large-volume DVT can restore venous patency and reduce the risk of VTE that results in pulmonary embolism (PE) (3–5).

Over the past decade, there has been a rapid increase in endovascular device options for the management of acute DVT, with the aim of enhanced thrombectomy efficiency while minimizing procedure-related complications (6). These catheter-directed interventions belong to either catheter-directed thrombolysis (CDT), or pharmacomechanical catheter-directed thrombolysis (PCDT) devices (7, 8). CDT is a method that delivers chemical pharmacological lysis directly into a thrombosed vascular segment using an intraluminal catheter (8, 9). The AngioJet Rheolytic Thrombectomy (ART; Boston Scientific, Marlborough, MA) is a PCDT device that utilizes mechanical fragmentation, chemical pharmacologic lysis, as well as rheolytic aspiration to remove intraluminal thrombus (10, 11). In thrombectomy mode, the device uses multiple high-velocity, high-pressure saline jets directed through the tip of an endovascular catheter to induce a localized low-pressure zone (Venturi-Bernoulli effect) (10, 12). This mechanism creates a localized vacuum effect, facilitating the dissociation of intravascular thrombus into smaller particles. The debris particles are then propelled proximally through an exhaust lumen for aspiration and removal. ART also has a Power Pulse mode, allowing for infusion of chemical pharmacological thrombolytics directly into the thrombosed vascular segment prior to the initiation of thrombectomy mode to further mechanically fragment and remove the thrombus (10).

Multiple clinical trials have investigated the clinical efficacy of ART in the management of acute DVT, with an established target lesion revascularization of 75%–100%, and venous patency of 68%–94% over 1 year (4, 7, 12, 13). However, previously a Food and Drug Administration (FDA) black box warning was issued on the use of ART due to a higher than anticipated risk of perioperative death. This warning followed reports of intraoperative fatalities during the treatment of pulmonary embolism, and was attributed to hemolysis-induced bradyarrhythmias, hemodynamic compromise, and distal embolization (8). There is wide clinical adoption of ART, and questions have remained about the potential mechanisms that contributed to intraoperative complications (8, 10, 13). Currently there is limited large animal data that has rigorously evaluated the efficacy, perioperative procedural outcomes, and histomorphic tissue level impact of ART. To address this, we used a large animal hyperacute DVT model in Yorkshire pigs to evaluate both the thrombectomy efficacy of ART as well as its impact on venous wall integrity.

## Materials and methods

### Animals

All animal procedures were conducted using an animal protocol that was approved by the Washington University in St. Louis School of Medicine Animal Studies Committee. All ethical and humane considerations under the National Institutes of Health guide for the care and use of Laboratory animals (NIH Publications No. 8023, revised 1978) were followed throughout our investigative pursuits.

Procedures were performed using adult, 96–113 kilograms, male Yorkshire pigs from Oak Hill Farm (Apple River, Illinois). For ethical considerations and to minimize large animal unnecessary suffering, the experimental sample size was minimized to the minimum necessary cohort of six animals (*n* = 3 per group). A formal power assessment was not feasible due to the novel nature of our planned large animal histomorphometric assessments and the absence of prior data on ART-induced venous microtrauma. Pigs were housed one pig per pen and allowed to acclimate for 72 h before the procedure. Pigs were given a regular chow diet and underwent an overnight fast prior to the procedure.

### Procedural preparation

Intravenous access is obtained in the ear pinna of the adult Yorkshire pigs. An anesthetic cocktail of Telazol 4 mg/kg, Ketamine 2 mg/kg, and Xylazine 2 mg/kg was administered to sedate the animal, place it in a supine position, secure limbs to the operating room table, and induce general anesthesia using isoflurane 1%–3% in 100% oxygen. Animals underwent endotracheal intubation and were maintained on ventilator support. Hemodynamics, including blood pressure and heart rate, were monitored following the induction of anesthesia. Oxygen saturation was also monitored using a pulse oximeter attached to the ear pinna. Respiratory rate and end-tidal CO_2_ were monitored using the respirator. Continuous electrocardiography was utilized throughout the procedure to monitor heart rate and rhythm. Animal vital signs were monitored throughout the procedure.

Using ultrasound guidance, the right femoral artery was percutaneously cannulated using an 18-gauge needle, and over a guidewire a 7Fr 11 cm Cordis Brite Tip sheath was placed in the femoral artery (Cordis, Miami Lakes, FL). The sheath was then attached to an arterial line for real-time monitoring of intra-operative blood pressure and systolic waveforms.

### Acute DVT formation

Large volume hyperacute DVT was induced in the infrarenal inferior vena cava (IVC) as previously described (14). This DVT model was chosen given its technical feasibility and to provide acute thrombus substrate that ART is reported to ideally suited to lyse and remove (11). Using ultrasound guidance, the left femoral vein was percutaneously cannulated, and over a guidewire the cannulation was dilated and a 12Fr 45 cm Ansel Sheath (Cook Medical, Bloomington, IN) was placed in the femoral vein. Over a 0.035″ 180 cm Amplatz stiff wire, a 32 mm CODA Balloon Catheter (Cook Medical, Bloomington, IN) was advanced through the sheath, was advanced to the distal IVC bifurcation using fluoroscopic guidance.

A longitudinal 5 cm incision was made in the left neck. Alternating blunt and sharp dissection was used to expose the left external jugular vein. The vein was cannulated by direct venous puncture using an 18G needle, and an 0.035″ Amplatz wire was then advanced into IVC using fluoroscopic guidance. A 12Fr 80 cm Ansel Sheath (Cook Medical, Bloomington, IN) was then advanced to the juxtarenal IVC. Another 32 mm CODA Balloon Catheter was advanced through the jugular sheath to a level directly below the renal veins.

**Figure 1:**
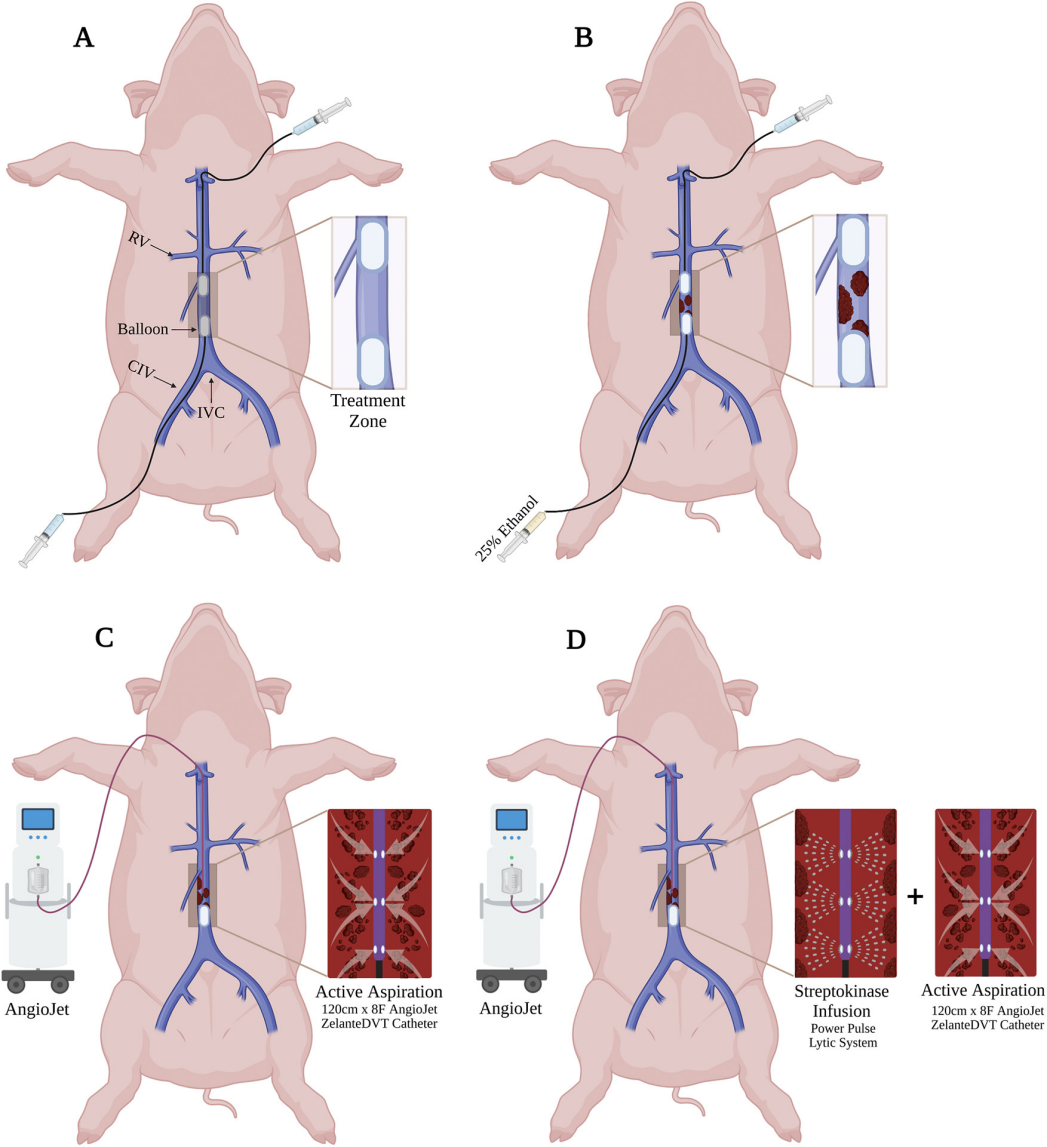
*In vivo* porcine acute DVT model and ART system. **(A)** Two 10Fr CODA Balloon Catheters were introduced into IVC through percutaneous left femoral venous access (distal balloon) and external right jugular venous access (proximal balloon). **(B)** The treatment zone was created by inflated endovascular balloons and intraluminal 25% ethanol administration in the static region. **(C)** For the mechanical thrombectomy, the proximal balloon was deflated, and 8Fr ART ZelanteDVT Catheter was advanced to the treatment zone under fluoroscopy, then active aspiration was activated. **(D)** For the pharmacomechanical catheter-directed thrombolysis (PCDT), the proximal balloon was deflated, and 8Fr ART ZelanteDVT Catheter was advanced to the treatment zone under fluoroscopy, then streptokinase was infused, then active aspiration was activated.

**Figure 2:**
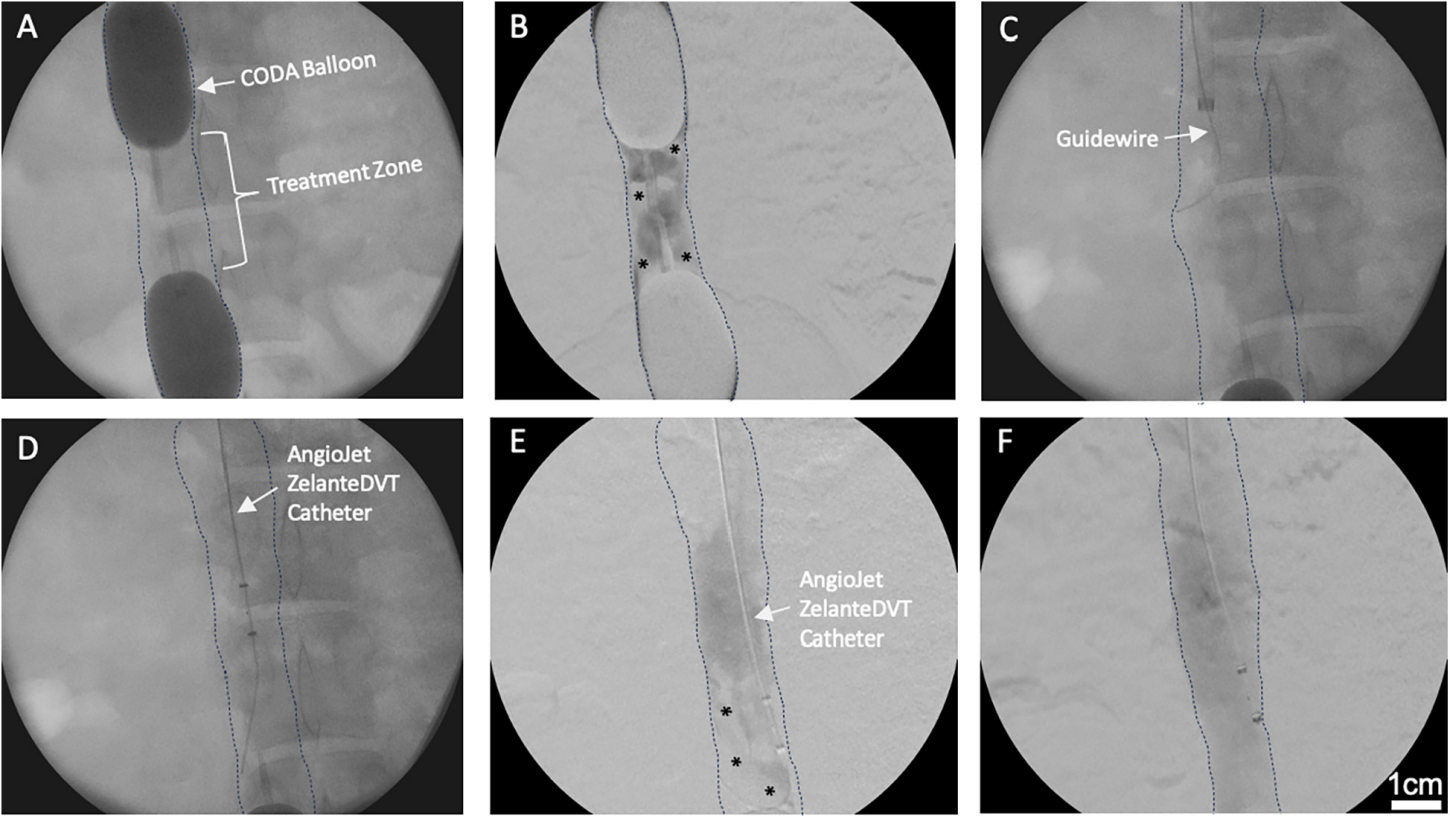
Venography of *in vivo* porcine acute DVT model and ART system. **(A)** Two CODA Balloon Catheters were inflated into IVC to create a treatment zone. **(B)** Thrombus (Asterisks) was developed within the treatment zone after 25% ethanol infusion. * indicates intraluminal thrombus. **(C)** Amplatz stiff guidewire was introduced into the treatment zone. **(D)** ART ZelanteDVT Catheter was introduced over the guidewire into the treatment zone. **(E)** Mechanical thrombectomy was initiated by activating the ART thrombectomy system. * indicates intraluminal thrombus. **(F)** Pharmacomechanical catheter-directed thrombolysis was initiated by injecting 50,000 IU 10 ml Streptokinase through ART Power Pulse, then ART console was activated.

Once in position, the proximal and distal CODA balloons were then inflated under fluoroscopy (Figures 1A, 2A). As previously described (14), through the femoral CODA Balloon Catheter, 15 ml of [25% ethanol and 75% normal saline (NS)] solution was slowly infused into the sequestered IVC segment. Acute DVT formation in the defined treatment zone was confirmed following 30 min of stasis induction (Figures 1B, 2A).

### ART mechanical and pharmacomechanical catheter-directed thrombolysis

A longitudinal 5 cm incision was made in the right neck. The right external jugular vein was exposed and directly cannulated. Over an 0.035″ Amplatz wire an 8Fr ART ZelanteDVT Catheter (Boston Scientific, Marlborough, MA) was advanced into the juxtarenal IVC. The jugular CODA balloon was deflated, while the femoral CODA balloon was kept inflated. The ART was advanced with care into the treatment zone (Figures 1C, 2D), connected to the ART console, and primed with a 0.9% 500 ml bag of normal saline per manufacturer instructions. For mechanical thrombectomy (MT) alone the ART catheter was advanced and retracted within the treatment zone at 1 mm per second over a 5 min period. Serial venograms through the inflated femoral CODA catheter were performed every 60 s during this treatment period.

For PCDT, a 0.9% 250 ml of normal saline supplemented with 10 ml of 50,000 IU of Streptokinase was used. After priming the ART console, Power Pulse mode was first used, and the ART ZelanteDVT Catheter was advanced and retracted over the wire within the treatment zone at 1 mm per second under fluoroscopic supervision for 5 min (Figure 2D). Following, pulsing the lytic action and thrombectomy aspiration mode was then initiated, and the ART was advanced and retracted in the treatment zone at 1 mm per second (Figures 1C, 2E). The segment was treated with MT over a 5 min period, with serial venograms performed every 60 s to assess residual thrombus volume (Figures 1D, 2F).

### Retroperitoneal exploration and IVC tissue harvest

**Figure 3:**
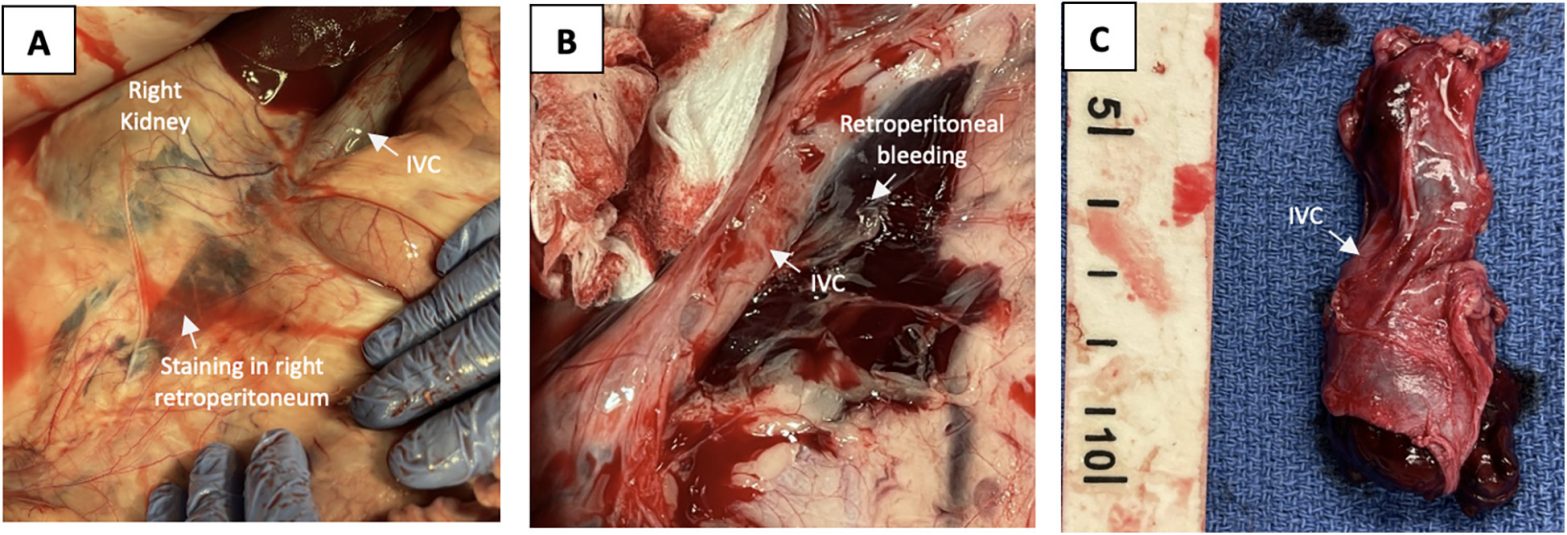
Intra-operative retroperitoneal exposure and gross pathology. **(A)** Right retroperitoneal open exposure of the IVC treatment zone with an inflated distal balloon. **(B)** Extensive retroperitoneal blood and tissue staining directly adjacent to the infrarenal IVC. **(C)** IVC treatment zone segment extracted *en bloc*.

A midline laparotomy was performed, followed by a left visceral rotation. A right retroperitoneal exposure was performed to visualize the infrarenal IVC (Figures 3A,B). The retroperitoneal fascia was excised longitudinally and the IVC, common iliac veins, and renal veins were exposed and controlled. 2-0 silk ties were used to tie off the sequestered segment (distal to the proximal CODA balloon and proximal to the distal CODA balloon). 88 mg/ml KCl was administered through and ear intravenous line to euthanize the pig. Following confirmed apnea, and asystole on monitor, the IVC balloons were deflated and removed, and the sequestered segment was immediately removed *en bloc*. The silk ties were removed, residual thrombus in the venous lumen was collected (Figure 3C). Both the IVC tissue and the intraluminal thrombus were then fixed in 10% formalin for histology assessments.

### Gross inspection and histopathologic evaluation

**Figure 4:**
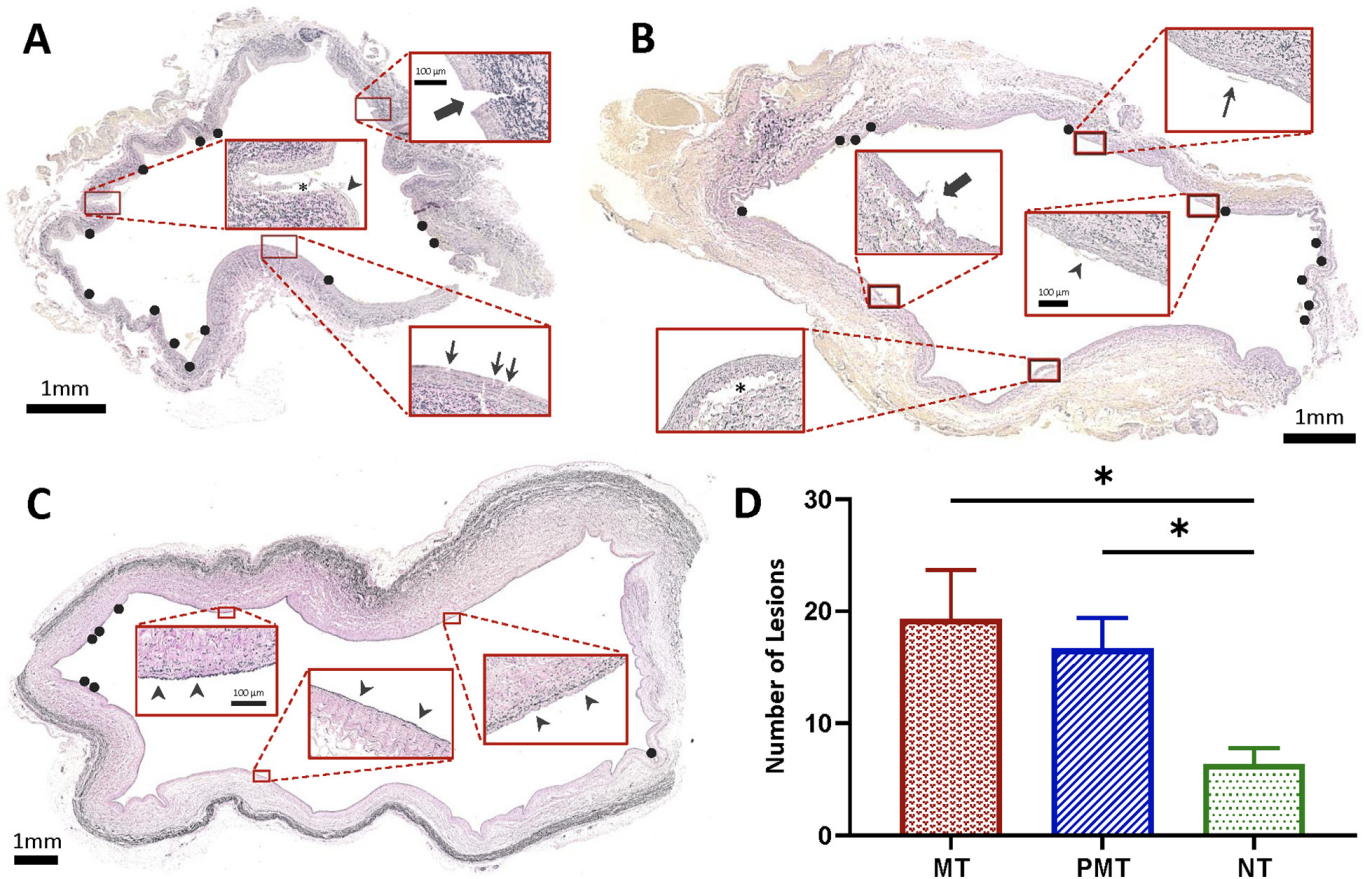
Histopathologic evaluation of IVC following MT and PCDT. **(A)** VVG-stained section of infrarenal IVC treated with mechanical thrombectomy (MT) demonstrating deep intimal disruption, extending beyond internal elastic lamina through media (thick arrow), superficial intimal tear (thin arrows), and detachment and elevation of the intima (asterisk) and endothelium (arrowhead). **(B)** VVG-stained section of (infrarenal) IVC treated with pharmacomechanical catheter-directed thrombolysis (PCDT) demonstrating separation of the intima and superficial media from the underlying layers (asterisk), disruption of intima (thick arrow), endothelial elevation (arrowhead), and detached endothelium (thin arrow). **(C)** VVG-stained section of non-treated suprarenal IVC with intact vessel wall (arrowheads). **(D)** Lesions in IVC specimens that were treated with either MT or PCDT compared to non-treated suprarenal IVC controls (*n* = 3 per group). Black circles show IVC wall lesions.

**Figure 5:**
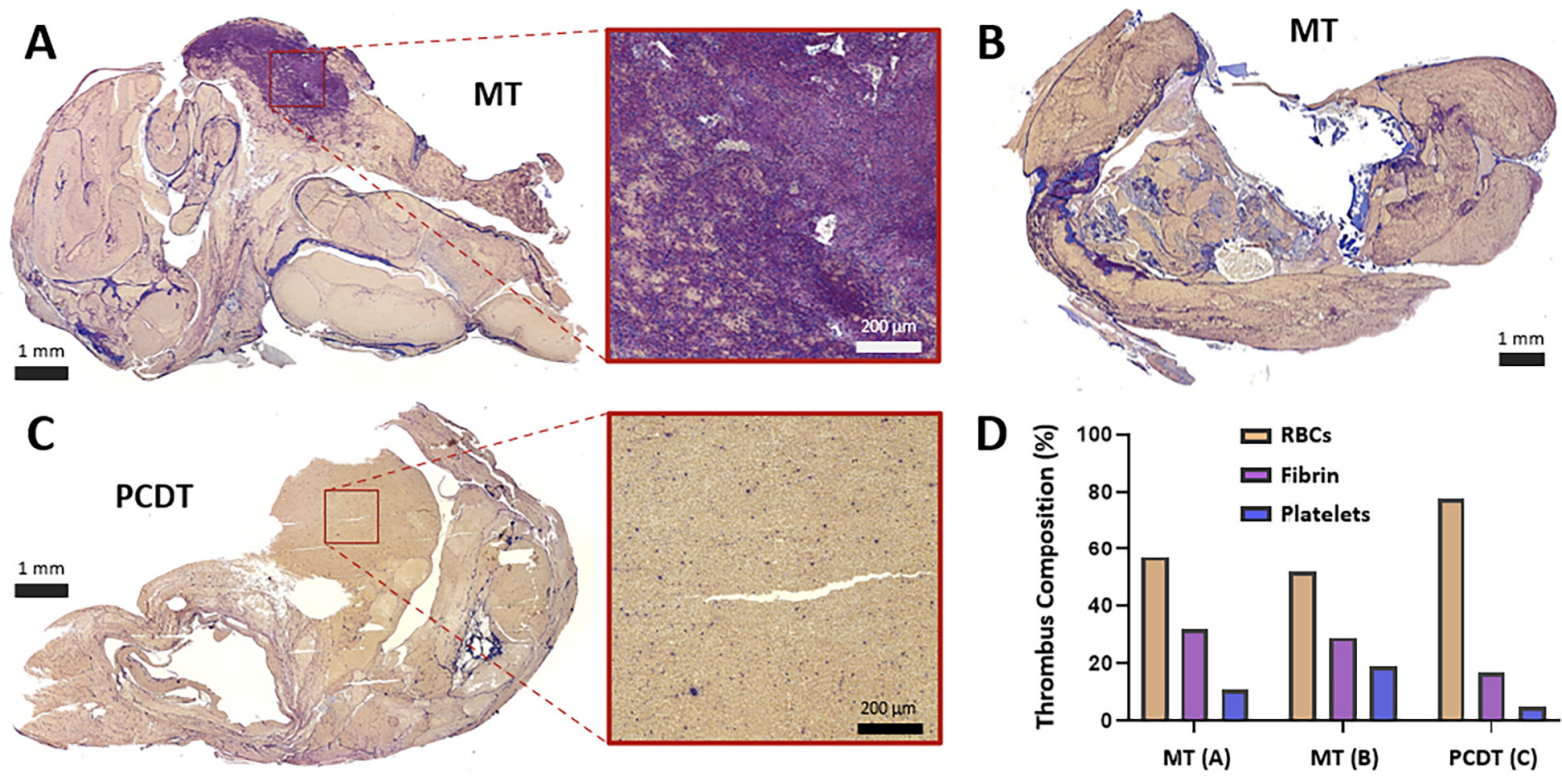
Histologic assessment of residual luminal thrombus composition following MT or PCDT. **(A)** Carstairs-stained residual thrombus after mechanical thrombectomy, with the representative region that is rich in thrombin (purple net-like structures) and platelets (blue fragments). **(B)** Carstairs-stained residual thrombus following mechanical thrombectomy. **(C)** Carstairs-stained residual thrombus after pharmacomechanical catheter-directed thrombolysis (PCDT), with representative red blood cell (RBC)-rich area (yellow staining. **(D)** Thrombus composition and quantification of RBC, fibrin, and platelet components of thrombi following MT and PCDT.

Gross inspection of the retroperitoneum revealed distinct staining patterns, which were qualitatively graded based on the extent of staining relative to the IVC. This grading system was specifically developed for the current study to establish a standardized method for assessing retroperitoneal trauma and bleeding—an outcome not previously characterized in the existing ART literature. This grading system assigned a score of 0 for “No Retroperitoneal Staining,” a score of 1 for “Limited Retroperitoneal Staining” indicating minimal to mild staining not directly covering the IVC, and a score of 2 for “Expanded Retroperitoneal Staining” denoting staining directly over and extensively around the IVC. Collected venous thrombus and IVC tissue, within the sequestered treatment zone and suprarenal IVC (control segment) were embedded in paraffin. Blocks of thrombus and tissue were then sectioned at 5 μm sections and mounted on microscope glass slides. Thrombus sections were stained with a modified Carstair method to evaluate content of red blood cells (RBC), platelets, and fibrin. Venous wall sections were stained with Hematoxylin and Eosin (H&E) and Verhoeff VanGieson (VVG) to evaluate wall architecture and screen for evidence of micro-trauma (intima disruption, elevation, or detachment; Figures 4A–C). Images of the stained thrombus and tissue stains were then captured using a NanoZoomer 2.0 slide scanner (Hamamatsu Photonics, Japan). ImageJ software was used to quantify the thrombus content of RBC, platelet, and fibrin components, and were reported as a percentage of the total area (Figures 5A–C).

### Statistical analysis

Pig vital signs were analyzed by a paired *t*-test with the Statistical Package for the Social Sciences (SPSS statistics version 25.0; SPSS, Inc, Chicago, IL), and all tests were two-sided with an alpha of 0.05.

## Results

### Pig survival and hemodynamics

All six pigs survived and tolerated the entire DVT formation and thrombectomy portions of the study. We observed no evidence of hemodynamic instability, incidence of arrhythmias, or major vascular complications. Placement of proximal and distal IVC occlusion balloons was uncomplicated and was tolerated with no hemodynamic changes in all pigs. Upon infusion of ethanol solution in the sequestered segments between the occlusion balloons, no significant changes in vital signs were observed (Table 1).

**Table 1:** Comparison of vital signs pre- and post-ethanol infusion.

Vital Sign	Pre-Ethanol	Post-Ethanol	*P*-value
Heart Rate	79 ± 13	77 ± 7	0.79
Respiratory Rate	13 ± 3	13 ± 2	0.56
Oxygen Saturation	100	100	0.99
End-tidal Carbon Dioxide	39 ± 1	40 ± 1	0.99

### Venography analysis

The average width and length of the sequestered venous segment in six pigs were 2.6 ± 0.6 and 5.5 ± 0.8 cm, respectively. Serial venograms in the sequestered segment demonstrated thrombus formation as early as 10 min post-ethanol infusion (PEI). Thrombus volume increased over time and reached 64.7% ± 9.7% maximal capacity thrombosis (MCT) at 30 min PEI. Overall, we observed that ART significantly decreased thrombus volume in all six pigs compared to baseline (64.7% ± 9.7% vs. 16.1% ± 10.5%; *P* *=* *0.02*; Figures 2E,F). In addition, thrombus reduction percentage was similar between MT and PCDT groups (75% ± 18% vs. 77% ± 13; *P* *=* *0.93*)*.*

### Gross inspection & histopathologic evaluation

ART caused traumatic wall injury in the treatment zone, leading to retroperitoneal staining in all six pigs (Figure 3B). In the grading system for retroperitoneal staining, the PCDT group had a higher mean cumulative score than the MT group (2 ± 0 vs. 1.3 ± 0.57; *P* *=* *0.11*). Compared to control segments, ART-treated segments demonstrated a significantly higher venous wall injury (18 ± 5.79 vs. 6.3 ± 2.51, *P* *=* *0.01* respectively; Figure 4D). No significant difference in vessel wall damage was detected between ART MT and PCDT-treated IVC segments (*P* *=* *0.63*). However, residual intraluminal thrombus composition differed in the two groups (Figure 5D). Quantification of the residual intaluminal thrombus composition revealed that the fibrin content was lower following PCDT compared to MT (17% vs. 29% and 17% vs. 32%; Figure 5D). One section per thrombus was obtained from the region demonstrating maximal cross-sectional area to ensure representative sampling and consistent analysis.

## Discussion

Our study evaluated the efficacy and safety of ART for the management of hyperacute large-volume DVT in a large animal. We observed that although ART can effectively remove venous thrombus using either MT or PCDT, post-procedural examination revealed evidence of venous wall trauma. No hemodynamic or vital sign changes were observed intraoperatively. However, open exposure of the retroperitoneum revealed tissue venous blood oozing, and gross evidence of venous wall microtrauma. Histopathologic assessment of residual intraluminal thrombus confirmed efficacy of adjunct lytics in reducing thrombus fibrin content.

Several studies have evaluated the efficacy of ART and its safety and complication profile in humans (4, 10, 15–17). In the Peripheral Use of ART with a Variety of Catheter Lengths (PEARL) registry, a total of 329 patients with lower extremity DVT underwent ART interventions, yielding successful outcomes with complete or significant (50%–99%) thrombus reduction observed in 96% of the patients (4). A meta-analysis of 12 clinical trials compared ART and CDT treatments for the management of lower extremity DVT. The study revealed no differences in thrombus removal, major adverse events, and symptom improvement between the two types of interventions (10).

Catheter-directed thrombolysis (CDT) has been extensively utilized for managing acute deep vein thrombosis (DVT), but its long-term efficacy in preventing post-thrombotic syndrome (PTS) remains uncertain. Several randomized controlled trials, including CaVenT, ATTRACT, and CAVA, have investigated the potential of CDT to prevent PTS development (9, 18, 19).

The CaVenT trial showed a significant reduction in PTS occurrence after 2 and 5 years, but this did not result in improved quality of life (9). The ATTRACT trial found no overall inpact on PTS prevention after 2 years, although a subgroup analysis of patients with iliofemoral DVT demonstrated lower symptom severity (18). Similarly, the CAVA trial, which included only iliofemoral DVT patients, did not find a significant preventive effect on PTS development or quality of life at 1 year (19). However, a *post hoc* analysis suggested that successful recanalization was associated with reduced symptom severity and quicker recovery of quality of life. Despite inconsistent evidence regarding long-term PTS prevention, CDT devices remain widely adopted due to their ability to rapidly restore venous patency and reduce acute thrombus burden. Additional studies are needed to clarify whether these short-term benefits translate into improved long-term outcomes, as PTS can develop many years after the acute thrombotic event (9, 18, 19).

Multiple studies have reported post-procedural complications following ART, including hemoglobinuria, pulmonary embolism, bradycardia, and acute kidney injury (10, 15, 16). In the treatment of pulmonary embolism, ART has been linked to major adverse events, including five intraoperative deaths (8). As a result, in 2007 the FDA issued a black box on ART to inform users of the potential risks associated with device use in the pulmonary arteries. There is limited pre-clinical data available regarding the safety profile of ART for management of DVT (20, 21). It is unknown how well high-pressure saline jets impact the venous wall during treatment of intraluminal DVT. It is reported that these high-pressure jets may cause bradycardia and hemoglobinuria due to red blood cell destruction (10, 15, 16). Hemolysis severity increases with the operation time length, leading to acute renal injury in severe cases (10, 15, 16).

Few studies have evaluated the thrombectomy efficacy and safety profile of ART in large animals, and none have conducted histomorphic analyses to evaluate venous wall integrity following treatment (20–22). Sharafuddin et al. conducted a study evaluating the safety and vascular integrity of the ART F105 catheter compared to the Fogarty balloon thrombectomy, utilizing a canine model (21). In this experimental model, the ART F105 catheter was introduced into the target vessel over a guidewire under fluoroscopic guidance, followed by activation in three back-and-forth cycles at a rate of 3–5 mm/sec along a 5 cm-long segment. Subsequently, the animals were euthanized, and the target vessel segments were harvested for gross inspection and histopathologic evaluation. The study findings revealed no significant difference in endothelial coverage or endothelial injury grade between the 15 ART-treated vessels (12 iliofemoral arteries and 3 iliac veins) and the 10 untreated vessels (8 arteries and 2 veins). Furthermore, the ART-treated vessels exhibited minimal focal endothelial denudation and no significant deep endothelial injury in comparison to the 4 Fogarty-treated vessels (3 iliofemoral arteries and 1 distal aorta) (21). This study's findings facilitated subsequent FDA regulatory approval (23). Of note, this study did not evaluate target vessel integrity in the setting of intraluminal thrombus. Additionally, they study did not use the Power Pulse mode, which in part could explain the differences observed when compared to our study.

A pre-clinical study compared the safety and thrombectomy efficacy of ART and ASPIRE thrombectomy devices in a bilateral iliac DVT pig model (22). This demonstrated that both devices had similar thrombectomy efficacy using intravascular ultrasound (IVUS) assessment. However, the ART group had significantly higher PE and hemolysis than the ASPIRE group. Scanning electron microscopy evaluation of the harvested vessels revealed a similar level of endothelial cell loss between groups (ART, 37.4% ± 16%; ASPIRE, 39.7% ± 24%) (22). Another pre-clinical study examined the structural integrity of vessel walls following ART MT and PCDT in an acute and chronic porcine iliac and carotid artery thrombosis model (20). The study revealed that both MT and PCDT had similar histologic injury grades. In the acute group, the level of endothelial denudation in the MT and PCDT vessels was 43% and 39%, respectively, while vessels with intact internal elastic lamina (IEL) in the MT and PCDT groups were 54% and 57%, respectively. In the chronic group, the level of ED in the MT and PCDT vessels was 52% and 49%, respectively, while vessels with IEL in the MT and PCDT groups were 38% and 32%, respectively (20). Our study demonstrated comparable results, indicating no significant difference in the extent of venous wall trauma between the MT and PCDT groups (19.3 ± 4.3 vs. 16.7 ± 2.7; *P* *=* *0.63*).

Our study demonstrated that ART had high thrombectomy efficacy, but it also appears to cause microvacular injuries to the treated venous segment. The impact of venous microtrauma is unclear, but can presumably be problematic in patients with DVT if it serves as a potential nidus for recurrent thrombus formation, re-occlusion, and need for possible reintervention (24). Indeed, the ATTRACT trial report a recurrent venous thromboembolism rate of approximately 12.5% at 24 months following PCDT (18). Although the precise etiology for this observed recurrence is unknown, it can potentially be attributed to altered venous anatomy, and thus more prone to re-thrombosis. The clinical implications of the venous microtrauma observed in our study warrant further investigation. Gumus and Arslanturk reported that balloon-assisted PCDT resulted in superior 12-month venous patency rates (84.3% for common iliac vein) compared to standard PCDT (51.6%), along with significantly lower post-thrombotic syndrome scores (25). Although their study focused on balloon dilation rather than ART specifically, the findings support the notion that procedural strategies minimizing venous wall trauma may enhance long-term outcomes. The focal venous microtrauma induced by ART in our model my predispose to thrombus re-formation or long-term scar tissue, which can compromise vessel patency and potentially contribute to the development of post-thrombotic syndrome. Of interest for future studies, is to evaluate whether the extent of acute venous wall injury correlates with long-term patency and clinical outcomes following ART.

Our study findings also diverse from prior reported preclinical studies, and we presume this is due to several important factors. First, in our study, we created venographically evident intraluminal thrombus in the IVC, and then activated Power Pulse and MT modes for a total of 10 min to replicate the clinical setting (20–22). In contrast, other studies evaluated the impact of ART for much shorter time periods (20). Second, our study used the contemporary ZelanteDVT catheter, whereas prior earlier studies used older device generations that may possibly have different performance characteristics.

We acknowledge several limitations of our study. First, the data were derived from a pre-clinical porcine model, which may not fully replicate the human coagulation cascade, acute deep vein thrombosis (DVT) pathophysiology, or venous wall architecture. Future investigations should explore alternative thrombus induction methods that better mimic human physiological conditions. Second, our model represents a hyperacute DVT scenario and does not capture subacute or chronic thrombus stages. This is clinically relevant, as most patients present with more organized thrombi that have evolved over days to weeks. Such thrombi, characterized by increased fibrin content and stronger adherence to the vessel wall (2), may require more aggressive mechanical forces for removal—potentially resulting in more severe venous injury than observed in our hyperacute model. Additionally, our sample size was limited to six animals [*n* = 3 for mechanical thrombectomy (MT) alone and *n* = 3 for Power Pulse + MT], and a formal power analysis was not feasible due to the novel nature of the histomorphometric assessments. Nevertheless, the consistent findings across all animals and the statistically significant differences observed support the robustness of our conclusions.

Another limitation of our study is the absence of a control group treated with alternative thrombectomy techniques, such as catheter-directed thrombolysis (CDT) without mechanical intervention or other mechanical thrombectomy devices. This design constraint limits our ability to attribute the observed venous wall microtrauma specifically to ART's high-pressure rheolytic mechanism, as opposed to thrombus removal procedures more broadly. Future comparative studies incorporating diverse thrombectomy modalities are needed to determine whether the venous injuries we observed are unique to ART's rheolytic technology or reflect a common consequence of endovascular thrombectomy.

All animals in this study were euthanized immediately post-procedure, which precluded evaluation of downstream sequelae associated with the observed venous microtrauma. Future studies should incorporate post-procedural observation periods to assess the clinical relevance of venous wall injury, including its impact on healing, vessel patency, and long-term outcomes. In addition, the use of animal models with longer thrombus indwelling times may more accurately reflect the subacute and chronic thrombus characteristics encountered in human DVT, thereby enabling a more comprehensive evaluation of device efficacy and associated injury risk.

The retroperitoneal staining grading system introduced in this study represents a novel, standardized tool for quantifying gross pathological changes not previously assessed in thrombectomy research. While external validation is needed, the system demonstrated reproducibility and practical utility in capturing procedure-related trauma. To better understand the long-term implications of ART-associated venous injury, future preclinical studies should employ our validated DVT model and grading system in larger cohorts. These studies could consider including thrombi of varying maturity (acute, subacute, and chronic) and incorporate comparisons of different thrombectomy technologies, with extended follow-up to evaluate venous remodeling, recurrent thrombosis, and long-term patency.

In conclusion, ART successfully reduces thrombus volume in a porcine acute DVT model in the infrarenal IVC. However, we observed evidence of post-procedure venous wall micro-trauma and retroperitoneal blood oozing. Histopathologic assessment of the harvested IVC segments revealed a significant increase in venous wall intimal tears for the IVC that was treated with ART. There was no significant difference observed in the MT and PCDT groups. Future *in vivo* experiments and clinical comparisons may need to investigate the safety, efficacy, and short- and long-term impact of venous microtrauma related to the use of ART.

## Data Availability

The raw data supporting the conclusions of this article will be made available by the authors, without undue reservation.
